# Combined effect of age and body mass index on postoperative mortality and morbidity in laparoscopic cholecystectomy patients

**DOI:** 10.3389/fsurg.2023.1243915

**Published:** 2023-11-23

**Authors:** Hana M. A. Fakhoury, Ziad Yousef, Hani Tamim, Sarah Daher, Abdul Aleem Attasi, Abdulaziz Al Ajlan, Ali H. Hajeer

**Affiliations:** ^1^ Department of Biochemistry and Molecular Medicine, College of Medicine, Alfaisal University, Riyadh, Saudi Arabia; ^2^ Department of Surgery, College of Medicine, King Saud bin Abdulaziz University for Health Sciences, Riyadh, Saudi Arabia; ^3^ Department of Surgery, King Abdulaziz Medical City, Riyadh, Saudi Arabia; ^4^ Department of Internal Medicine, American University of Beirut Medical Center, Beirut, Lebanon; ^5^ Department of Anesthesia, King Abdullah Specialized Children Hospital, Riyadh, Saudi Arabia; ^6^ Department of Pathology and Laboratory Medicine, King Abdulaziz Medical City, Riyadh, Saudi Arabia

**Keywords:** age, laparoscopic cholecystectomy, BMI (Body mass index), ACS NSQIP, obesity paradox

## Abstract

**Background:**

Previous studies have assessed the impact of age and body mass index (BMI) on surgery outcomes separately. This retrospective cohort study aimed to investigate the combined effect of age and BMI on postoperative mortality and morbidity in patients undergoing laparoscopic cholecystectomy.

**Methods:**

Data from the American College of Surgeons National Surgical Quality Improvement Program (ACS NSQIP) database for laparoscopic cholecystectomy patients between 2008 and 2020 were analyzed. Patient demographics, functional status, admission sources, preoperative risk factors, laboratory data, perioperative variables, and 30-day postoperative outcomes were included in the dataset. Logistic regression was used to determine the association of age, BMI, and age/BMI with mortality and morbidity. Patients were stratified into different subcategories based on their age and BMI, and the age/BMI score was calculated. The chi-square test, independent sample *t*-test, and ANOVA were used as appropriate for each category.

**Results:**

The study included 435,052 laparoscopic cholecystectomy patients. Logistic regression analysis revealed that a higher age/BMI score was associated with an increased risk of mortality (adj OR 13.13 95% CI, 9.19–18.77, *p* < 0.0001) and composite morbidity (adj OR 2.57, 95% CI 2.23–2.95, *p* < 0.0001).

**Conclusion:**

Older age, especially accompanied by a low BMI, appears to increase the post-operative mortality and morbidity risks in laparoscopic cholecystectomy patients, while paradoxically, a higher BMI seems to be protective. Our hypothesis is that a lower BMI, perhaps secondary to malnutrition, can carry a greater risk of surgery complications for the elderly. Age/BMI is strongly and positively associated with mortality and morbidity and could be used as a new scoring system for predicting outcomes in patients undergoing surgery. Nevertheless, laparoscopic cholecystectomy remains a very safe procedure with relatively low complication rates.

## Introduction

Older age has been found to be associated with an increased risk of mortality following surgery. For instance, previous studies have reported that age is the strongest predictor of mortality in patients with bacterial colitis undergoing emergency colectomies, with a nine-fold risk increase in older patients (1). Additionally, older age has been identified as a risk factor for death in super-obese individuals having bariatric surgery (2). On the other hand, BMI has been found to be negatively associated with mortality (3). In contrast, Mullen et al. (4) investigated the effect of BMI on patients undergoing major intra-abdominal cancer surgery. Their results demonstrated that obesity is not a risk factor for postoperative mortality or major complications. Interestingly, they found that being underweight carried a fivefold increased risk of postoperative mortality. However, the effect of age and BMI on surgery outcomes in combination has not been well studied.

Our group introduced a new scoring system consisting of the ratio of age over BMI (age/BMI), referred to as the Hajeer score, as a predictor of mortality in COVID-19 patients (5).This score was found to be a stronger predictor of death compared to age alone (5).

Laparoscopic cholecystectomy is a relatively low-risk procedure with a large sample size, as laparoscopic biliary procedures are associated with lower overall morbidity and shorter hospital stays than open surgery (6). The aim of this study was to investigate the combined effect of age and BMI on postoperative morbidity and mortality in laparoscopic cholecystectomy patients.

## Materials and methods

### Study design and data collection

We used a retrospective cohort study design to analyze data from the American College of Surgeons National Surgical Quality Improvement Program (ACS NSQIP) database. This database is a prospective, validated outcomes registry that provides feedback to member hospitals on 30-day risk-adjusted surgical mortality and morbidity. It contains anonymized data for patients undergoing major surgery in over 700 participating non-Veterans Affairs administration hospitals. The data includes patient demographics, functional statuses, admission sources, preoperative risk factors, laboratory data, perioperative variables, and 30-day postoperative outcomes. Trained surgical clinical reviewers collect this data on admission from the medical chart, operative log, anesthesia record, interviews with the surgical attending, and telephone interviews with patients (7). For this study, we identified 435,052 patients who underwent laparoscopic cholecystectomy between 2008 and 2020 using the Current Procedural Terminology (CPT) code 47,562 maintained by the American Medical Association.

The King Abdullah International Medical Research Center's Institutional Review Board (IRB) of the Ministry of National Guard—Health Affairs in Riyadh, Saudi Arabia waived the ethical approval and requirement of a consent form. All methods were conducted following the relevant guidelines and regulations, and all data were de-identified before analysis.

### Subgroups

Age, BMI, and age/BMI were converted from continuous to categorical variables in order to perform an adjusted multivariable risk assessment for each. Age was divided into four groups, namely group 1 (<40 years), group 2 (40– < 60 years), group 3 (60– < 80 years), and group 4 (>80 years). On the other hand, BMI was divided into three groups, namely group 1 (BMI < 25), group 2 (BMI 25– < 30), and group 3 (BMI > 30). Age/BMI score was calculated by dividing age (years) by BMI (kg/m2). The range for age/BMI in this cohort was min = 0.22, max = 8.42, 25th percentile** **= 1.08, and 75th percentile = 2.12. Age/BMI was divided into four groups, namely group 1 (age/BMI < 1.0), group 2 (age/BMI > 1.0– < 2.0), group 3 (age/BMI > 2.0–<3.0), and group 4 (age/BMI > 3.0). Finally, to test our results, we divided the patients into nine groups according to age and BMI before analyzing the incidence of mortality and morbidity. These groups were as follows: Group 1 (age < 50 and BMI < 18.5), group 2 (age < 50 and BMI 18.5–25), group 3 (age < 50 and BMI > 25), group 4 (age 50–70 and BMI < 18.5), group 5 (age 50–70 and BMI 18.5–25), group 6 (age 50–70 and BMI > 25), group 7 (age > 70 and BMI < 18.5), group 8 (age > 70 and BMI 18.5–25), and group 9 (age > 70 and BMI > 25).

### Extracted variables

The demographic data obtained from patients included their age, sex, height, weight, BMI, and race. The comorbidities and characteristics of the patients assessed included obesity, smoking, diabetes, hypertension, chronic steroid use, systemic sepsis (within 48 h prior to surgery), dyspnea, congestive heart failure (within 30 days prior to surgery), severe chronic obstructive pulmonary disease (COPD), ascites (within 30 days prior to surgery), acute renal failure (preoperative), bleeding disorders, and disseminated cancer. The operative characteristics included full operation (OP) time, inpatient/outpatient status, American Society of Anesthesiologists (ASA) classification (ASA scores: I is a healthy patient; II is mild systemic disease but no functional limitations; III is severe systemic disease with definite functional limitations; IV is severe systemic disease that is a constant threat to life; and V is a moribund patient unlikely to survive 24 h with or without an operation), and preoperative blood transfusion (within 72 h prior to surgery).

The following outcomes were assessed in this study: mortality, wound outcome, cardiac outcome, respiratory outcome, urinary outcome, central nervous system (CNS) outcome, thromboembolism, sepsis, return to operation room (OR), and composite morbidity. Composite morbidity was defined as the presence of any of the other nine outcomes.

### Statistical analysis

Descriptive statistics were reported as numbers and percentages for categorical variables, and mean and standard deviation for continuous variables. Associations between exposure (age, BMI, and age/BMI) and the different characteristics and outcomes were assessed using the chi-square test for categorical factors, the independent samples t-test for continuous factors in association with a 2-group categorical variable, or ANOVA for continuous factors in association with a 3-group or more categorical variable.

To control for potentially confounding effects of patient characteristics, we conducted multivariate logistic regression analyses. Adjusted odds ratios (aORs) for mortality and morbidities were calculated using logistic regression with 95% confidence intervals (CIs). Variables considered in the multivariate analysis were: underlying medical conditions [congestive heart failure within 30 days prior to surgery, severe COPD, acute renal failure (pre-operative), bleeding disorders, cancer, ascites within 30 days prior to surgery], preoperative treatments (dialysis, ventilation, chemotherapy, radiation therapy), and ASA classification (I/II, III, and IV/V). The level of significance for the *p*-value was set at <0.05.

## Results

A total of 435,052 patients who had undergone laparoscopic cholecystectomy were included in this study, of which 11.1% were emergency cases. Supplementary Table S1 in the supplement describes the demographics of the four groups divided by age. The results indicated that as age increased, weight, BMI, the proportion of female gender, and smoking decreased. On the other hand, as age increased the following increased: OP in minutes, white race, the prevalence of BMI > 30, diabetes, hypertension, systemic sepsis, steroid use, ASA IV/V, being an in-patient, requirement for transfusion, wound infection, dyspnea, congestive heart failure, COPD, ascites, renal failure, bleeding disease, and disseminated cancer.

Table 1 describes the demographics of the four age/BMI groups. Except for female representation, BMI, and smoking, all other comorbidities increased as the age/BMI score increased.

**Table 1:** Baseline characteristics of the age/BMI categories. Results are presented as mean (SD) for continuous variables and *N* (number) and % for categorical variables. Age/BMI was divided into four groups: group 1 (age/BMI < 1.0), group 2 (age/BMI > 1.0 & <2.0), group 3 (age/BMI > 2.0 & <3.0), and group 4 (age/BMI > 3.0).^a^

	Age/BMI Groups				
	Group 1	Group 2	Group 3	Group 4	*P* value
Number	89,130	2,17,192	1,04,635	24,095	
Age Mean (SD)	29.05 (7.33)	46.87 (11.31)	65.56 (9.65)	77.89 (8.27)	<0.0001
Sex (female) *N* (%)	75,481 (84.72)	1,53,932 (70.91)	61,194 (58.52)	14,414 (59.87)	<0.0001
Height (inch) Mean (SD)	64.47 (3.73)	65.09 (3.95)	65.44 (4.04)	64.98 (4.07)	<0.0001
Weight (lb) Mean (SD)	226.95 (62.16)	193.57 (44.82)	167.86 (31.00)	137.34 (25.17)	<0.0001
BMI Mean (SD)	38.25 (9.10)	32.03 (6.47)	27.47 (3.90)	22.76 (2.91)	<0.0001
Age/BMI	0.77 (0.15)	1.48 (0.28)	2.40 (0.27)	3.46 (0.44)	<0.0001
OP time (min) Mean (SD)	68.54 (43.64)	69.25 (48.08)	72.42 (56.53)	73.25 (56.13)	<0.0001
Race (white) *N* (%)	59,193 (66.41)	1,47,771 (68.04)	73,599 (70.34)	17,580 (72.96)	<0.0001
BMI > 30 *N* (%)	35,591 (24.91)	46,288 (28.72)	37,860 (33.94)	7,373 (37.96)	<0.0001
Smoking *N* (%)	18,272 (20.50)	38,810 (17.87)	14,426 (13.79)	2,432 (10.09)	<0.0001
Diabetes *N* (%)	5,258 (5.90)	26,973 (12.42)	19,153 (18.30)	3,892 (16.15)	<0.0001
Hypertension *N* (%)	10,189 (11.43)	66,469 (30.60)	55,536 (53.08)	15,346 (63.69)	<0.0001
Systemic sepsis in the previous 48 h *N* (%)	8,599 (6.02)	10,710 (6.64)	10,759 (9.65)	3,003 (15.46)	<0.0001
Steroid use *N* (%)	1,409 (0.99)	3,324 (2.06)	3,596 (3.22)	678 (3.49)	<0.0001
ASA IV/V *N* (%)	870 (0.98)	3,258 (1.50)	4,021 (3.85)	1,924 (8.00)	<0.0001
Inpatient *N* (%)	33,092 (37.13)	79,461 (36.59)	45,148 (43.15)	13,352 (55.41)	<0.0001
Transfusion *N* (%)	101 (0.11)	357 (0.16)	315 (0.30)	173 (0.72)	<0.0001
Wound infection *N* (%)	1,270 (1.42)	6,175 (2.84)	5,680 (5.43)	1,698 (7.05)	<0.0001
Dyspnea *N* (%)	2,423 (2.72)	8,022 (3.69)	5,938 (5.67)	1,922 (7.98)	<0.0001
Congestive heart failure in the previous 30 days *N* (%)	104 (0.12)	764 (0.35)	909 (0.87)	424 (1.76)	<0.0001
COPD *N* (%)	328 (0.37)	3,951 (1.82)	4,839 (4.62)	1,933 (8.02)	<0.0001
Ascites *N* (%)	45 (0.05)	246 (0.11)	248 (0.24)	89 (0.37)	<0.0001
Renal failure *N* (%)	32 (0.04)	277 (0.13)	306 (0.29)	91 (0.38)	<0.0001
Bleeding disease *N* (%)	711 (0.080)	3,809 (1.75)	4,634 (4.43)	1,714 (7.11)	<0.0001
Disseminated cancer *N* (%)	69 (0.08)	941 (0.43)	1,385 (1.32)	473 (1.96)	<0.0001

^a^ OP time, operation time; ASA, American Society of Anesthesiologists; COPD, chronic obstructive pulmonary disease.

Table 2 shows the results of the ordered regression analysis of the age groups with the different outcomes. Older age significantly predicted morbidity and mortality. Adjusted OR for the >80 years old were as follows: (OR 16.06 95% CI, 11.48–22.45, *p* < 0.0001), wound outcome (OR 1.91, 95% CI 1.53–2.38, *p* < 0.0001), cardiac outcome (OR 7.04, 95% CI 4.04–12.27, *p* < 0.0001), respiratory outcome (OR 2.27, 95% CI 1.81–2.86, *p* < 0.0001), urinary outcome (OR 2.56, 95% 1.57–4.19, *p* = 0.0002), CNS outcome (OR 13.62, 95% CI 5.23–35.50, *p* < 0.0001), thromboembolism (OR 2.30, 95% CI 1.62–3.27, *p* < 0.0001), sepsis (OR 2.28, 95% CI 1.88–2.77, *p* < 0.0001), return to OR (OR 1.21, 95% CI 0.99–1.19, *p* = 0.0647), and composite morbidity (OR 2.35, 95% CI 2.07–2.67, *p* < 0.0001).

**Table 2:** Effect of age on outcome. Results are presented as adjusted and unadjusted OR for each outcome. Age groups 40–<60, 60–<80, and >80 years old were compared to the reference group <40 years old.^a^

	Age Groups			
Outcome	<40 years	40–<60 years	60–<80 years	>80 years
Mortality *N* (%)	41 (0.03)	176 (0.11)	463 (0.42)	397 (2.04)
OR Unadj	Reference	3.81 (2.71–5.35)*	14.52 (10.55–19.99)*	72.71 (52.70–100.32)*
OR Adj	Reference	2.20 (1.56–3.10)*	4.66 (3.35–6.47)*	16.06 (11.48–22.45)*
Wound outcome *N* (%)	530 (0.37)	1,191 (0.74)	1,719 (1.54)	383 (1.97)
OR Unadj	Reference	2.00 (1.81–2.22)*	4.21 (3.81–4.64)*	5.40 (4.73–6.17)*
OR Adj	Reference	1.53 (1.28–1.83)*	2.13 (1.79–2.53)*	1.91 (1.53–2.38)*
Cardiac outcome *N* (%)	32 (0.02)	170 (0.11)	512 (0.46)	223 (1.15)
OR Unadj	Reference	4.71 (3.23–6.88)*	20.59 (14.40–29.43)*	51.86 (35.79–75.14)*
OR Adj	Reference	2.15 (1.23–3.77)**	4.53 (2.66–7.73)*	7.04 (4.04–12.27)*
Respiratory outcome N (%)	337 (0.24)	786 (0.49)	1,587 (1.42)	642 (3.31)
OR Unadj	Reference	2.07 (1.82–2.36)*	6.11 (5.43–6.87)*	14.46 (12.66–16.51)*
OR Adj	Reference	1.13 (1.15–1.78)**	2.02 (1.64–2.48)*	2.27 (1.81–2.86)*
Urinary outcome *N* (%)	54 (0.04)	223 (0.14)	449 (0.40)	153 (0.79)
OR Unadj	Reference	3.67 (2.72–4.93)*	10.69 (8.06–14.18)*	21.01 (15.40–28.66)*
OR Adj	Reference	2.79 (1.77–4.41)*	2.77 (1.76–4.35)*	2.56 (1.57–4.19)**
CNS outcome *N* (%)	11 (0.01)	36 (0.02)	105 (0.09)	78 (0.40)
OR Unadj	Reference	2.90 (1.48–5.70)**	12.24 (6.58–22.78)*	52.38 (27.86–98.49)*
OR Adj	Reference	1.80 (0.65–4.99)	5.36 (2.11–13.65)**	13.62 (5.23–35.50)*
Thromboembolism *N* (%)	196 (0.14)	358 (0.22)	477 (0.43)	156 (0.80)
OR Unadj	Reference	1.62 (1.36–1.93)*	3.13 (2.65–3.69)*	5.90 (4.78–7.28)*
OR Adj	Reference	1.35 (0.99–1.84)	1.82 (1.35–2.45)*	2.30 (1.62–3.27)*
Sepsis *N* (%)	476 (0.33)	1,201 (0.75)	2,170 (1.95)	719 (3.70)
OR Unadj	Reference	2.25 (2.02–2.50)*	5.94 (5.37–6.56)*	11.50 (10.235–12.93)*
OR Adj	Reference	1.72 (1.45–2.05)*	2.39 (2.02–2.82)*	2.28 (1.88–2.77)*
Return to OR *N* (%)	979 (0.69)	1,391 (0.86)	1,629 (1.46)	371 (1.91)
OR Unadj	Reference	1.26 (1.16–1.37)*	2.15 (1.98–2.33)*	2.82 (2.50–3.19)*
OR Adj	Reference	1.12 (0.96–1.30)	1.36 (1.16–1.58)*	1.21 (0.99–1.19)
Composite morbidity *N* (%)	1,330 (0.93)	3,039 (1.89)	5,096 (4.457)	1,708 (8.79)
OR Unadj	Reference	2.05 (1.92–2.18)*	5.10 (4.80–5.42)*	10.26 (9.54–11.05)*
OR Adj	Reference	1.54 (1.38–1.72)*	2.11 (1.89–2.35)*	2.35 (2.07–2.67)*

^a^ Variables considered and adjusted in the multivariate analysis were: underlying medical conditions [congestive heart failure within 30 days prior to surgery, severe COPD, acute renal failure (pre-operative), bleeding disorders, cancer, ascites within 30 days prior to surgery], preoperative treatments (dialysis, ventilation, chemotherapy, radiation therapy, and ASA classification.

* *p* < 0.0001.

** *p* < 0.05.

Supplementary Table S2 in the supplement describes the demographics of the three BMI groups. Factors inversely correlated with BMI included: Age, OP time, smoking, steroid, transfusion use, COPD, ascites, bleeding disease, and disseminated cancer. While, diabetes, hypertension, systemic sepsis in the previous 48hr, and dyspnea were positively correlated with increasing BMI.

Table 3 shows the results of the ordered regression analysis of the BMI groups with different outcomes. Except for thromboembolism and urinary outcomes, higher BMI was protective against mortality and morbidity. The adjusted odds ratios (OR) for BMI > 30 were as follows: mortality (OR 0.39, 95% CI 0.33–0.45, *p* < 0.0001), wound outcome (OR 0.74, 95% CI 0.65–0.84, *p* < 0.0001), cardiac outcome (OR 0.57, 95% CI 0.45–0.71, *p* < 0.0001), respiratory outcome (OR 0.85, 95% CI 0.75–0.97, *p* = 0.0160), CNS outcome (OR 0.46, 95% CI 0.30–0.70, *p* = 0.0004), sepsis (OR 0.81, 95% CI 0.72–0.90, *p* = 0.0002), return to OR (OR 0.74, 95% CI 0.65–0.84, *p* < 0.0001), and composite morbidity (OR 0.79, 95% CI 0.74–0.86, *p* < 0.0001).

**Table 3:** Effect of BMI on outcome. Results are presented as adjusted and unadjusted OR for each outcome. BMI group 2 (BMI > 25 & <30) and group 3 (BMI > 30) were compared to the reference group 1 (BMI < 25).^a^

	BMI Groups		
Outcome	<25	>25–<30	>30
Mortality *N* (%)	360 (0.46)	303 (0.24)	414 (0.18)
OR Unadj	Reference	0.52 (0.44–0.60)*	0.39 (0.34–0.45)*
OR Adj	Reference	0.60 (0.51–0.70)*	0.39 (0.33–0.45)*
Wound outcome *N* (%)	748 (0.95)	1,200 (0.94)	1,875 (0.82)
OR Unadj	Reference	0.99 (0.90–1.09)	0.86 (0.79–0.93)**
OR Adj	Reference	0.93 (0.82–1.07)	0.74 (0.65–0.84)*
Cardiac outcome *N* (%)	231 (0.29)	306 (0.24)	400 (0.17)
OR Unadj	Reference	0.82 (0.69–0.97)**	0.59 (0.50–0.70)*
OR Adj	Reference	0.79 (0.62–1.00)**	0.57 (0.45–0.71)*
Respiratory outcome *N* (%)	736 (0.94)	985 (0.77)	1,631 (0.71)
OR Unadj	Reference	0.82 (0.75–0.90)*	0.76 (0.69–0.82)*
OR Adj	Reference	0.99 (0.86–1.14)	0.85 (0.75–0.97)**
Urinary outcome *N* (%)	141 (0.18)	235 (0.18)	503 (0.22)
OR Unadj	Reference	1.03 (0.83–1.27)	1.22 (1.01–1.47)**
OR Adj	Reference	1.00 (0.76–1.32)	1.23 (0.96–1.57)
CNS outcome *N* (%)	68 (0.09)	71 (0.06)	91 (0.04)
OR Unadj	Reference	0.64 (0.46–0.90)**	0.46 (0.33–0.63)*
OR Adj	Reference	0.57 (0.36–0.91)**	0.46 (0.30–0.70)**
Thromboembolism *N* (%)	207 (0.26)	313 (0.25)	667 (0.29)
OR Unadj	Reference	0.93 (0.78–1.11)	1.10 (0.94–1.29)
OR Adj	Reference	0.96 (0.75–1.24)	1.06 (0.85–1.33)
Sepsis *N* (%)	923 (1.18)	1,399 (1.10)	2,244 (0.98)
OR Unadj	Reference	0.93 (0.86–1.02)	0.83 (0.77–0.89)*
OR Adj	Reference	0.94 (0.84–1.06)	0.81 (0.72–0.90)**
Return to OR *N* (%)	962 (1.23)	1,295 (1.02)	2,113 (0.92)
OR Unadj	Reference	0.82 (0.76–0.90)*	0.75 (0.69–0.81)*
OR Adj	Reference	0.88 (0.77–1.00)	0.74 (0.65–0.84)*
Composite morbidity *N* (%)	2,256 (2.88)	3,322 (2.61)	5,595 (2.44)
OR Unadj	Reference	0.91 (0.86–0.96)**	0.84 (0.80–0.89)*
OR Adj	Reference	0.91 (0.84–0.99)	0.79 (0.74–0.86)*

^a^ Variables considered and adjusted in the multivariate analysis were: underlying medical conditions [congestive heart failure within 30 days prior to surgery, severe COPD, acute renal failure (pre-operative), bleeding disorders, cancer, ascites within 30 days prior to surgery], preoperative treatments (dialysis, ventilation, chemotherapy, radiation therapy, and ASA classification.

* *p* < 0.0001.

** *p* < 0.05.

Table 4 shows the results of the ordered regression analysis of the age/BMI groups with different outcomes. A higher age/BMI score appears to be a risk for all studied outcomes. The adjusted OR for age/BMI group 4 (age/BMI > 3.0) were as follows: mortality (OR 13.13 95% CI, 9.19–18.77, *p* < 0.0001), wound outcome (OR 2.68, 95% CI 2.11–3.40, *p* < 0.0001), cardiac outcome (OR 8.66, 95% CI 4.36–17.19, *p* < 0.0001), respiratory outcome (OR 2.31, 95% CI 1.80–2.96, *p* < 0.0001), urinary outcome (OR 1.27, 95% CI 0.79–2.04, *p* = 0.3352), CNS outcome (OR 7.66, 95% CI 2.98–19.70, *p* < 0.0001), thromboembolism (OR 1.92, 95% CI 1.31–2.82, *p* = 0.0008), sepsis (OR 2.51, 95% CI 2.03–3.10, *p* < 0.0001), return to OR (OR 1.55, 95% CI 1.26–1.91, *p* < 0.0001), and composite morbidity (OR 2.57, 95% CI 2.23–2.95, *p* < 0.0001).

**Table 4:** Effect of age/BMI on outcome. Results are presented as adjusted and unadjusted OR for each outcome. Age/BMI group 2 (age/BMI > 1.0 & <2.0), group 3 (age/BMI > 2.0 & <3.0), and group 4 (age/BMI > 3.0) were compared to the reference group 1 (age/BMI < 1.0).^a^

	Age/BMI Groups			
Outcome	Group 1	Group 2	Group 3	Group 4
Mortality *N* (%)	35 (0.04)	235 (0.11)	428 (0.41)	379 (1.57)
OR Unadj	Reference	2.76 (1.93–3.93)*	10.46 (7.41–14.76)*	40.68 (28.77–57.53)*
OR Adj	Reference	2.26 (1.57–3.24)*	5.01 (3.52–7.13)*	13.13 (9.19–18.77)*
Wound outcome *N* (%)	361 (0.41)	1,555 (0.72)	1,440 (1.38)	467 (1.94)
OR Unadj	Reference	1.77 (1.58–1.99)*	3.43 (3.06–3.85)*	4.86 (4.23–5.58)*
OR Adj	Reference	1.84 (1.49–2.27)*	2.45 (1.98–3.02)*	2.68 (2.11–3.40)*
Cardiac outcome *N* (%)	20 (0.02)	261 (0.12)	438 (0.42)	218 (0.90)
OR Unadj	Reference	5.36 (3.40–8.45)*	18.73 (11.96–29.32)*	40.68 (25.73–64.32)*
OR Adj	Reference	3.43 (1.74–6.78)**	5.93 (3.03–11.63)*	8.66 (4.36–17.19)*
Respiratory outcome *N* (%)	254 (0.28)	1,121 (0.52)	1,348 (1.29)	629 (2.61)
OR Unadj	Reference	1.82 (1.58–2.08)*	4.57 (3.99–5.22)*	9.38 (8.10–10.86)*
OR Adj	Reference	1.61 (1.27–2.03)*	2.05 (1.62–2.59)*	2.31 (1.80–2.96)*
Urinary outcome *N* (%)	56 (0.06)	340 (0.16)	373 (0.36)	110 (0.46)
OR Unadj	Reference	2.49 (1.88–3.31)*	5.69 (4.30–10.07)*	7.30 (5.29–10.07)*
OR Adj	Reference	1.74 (1.14–2.66)**	1.74 (1.13–2.66)**	1.27 (0.79–2.04)
CNS outcome *N* (%)	9 (0.01)	54 (0.02)	95 (0.09)	72 (0.30)
OR Unadj	Reference	2.46 (1.22–4.98)**	8.99 (4.54–17.82)*	29.66 (14.84–59.32)*
OR Adj	Reference	1.54 (0.59–4.02)	3.34 (1.32–8.47)**	7.66 (2.98–19.70)*
Thromboembolism *N* (%)	154 (0.17)	483 (0.22)	402 (0.38)	148 (0.61)
OR Unadj	Reference	1.29 (1.07–1.54)**	2.23 (1.85–2.68)*	3.57 (2.85–4.48)*
OR Adj	Reference	1.42 (1.02–1.99)**	1.72 (1.22–2.41)**	1.92 (1.31–2.82)**
Sepsis *N* (%)	347 (0.39)	1,689 (0.78)	1,817 (1.74)	713 (2.96)
OR Unadj	Reference	2.01 (1.79–2.25)*	4.52 (4.03–5.07)*	7.80 (6.86–8.88)*
OR Adj	Reference	1.78 (1.47–2.16)*	2.25 (1.85–2.72)*	2.51 (2.03–3.10)*
Return to OR *N* (%)	637 (0.71)	1,869 (0.86)	1,386 (1.32)	478 (1.98)
OR Unadj	Reference	1.21 (1.10–1.32)*	1.87 (1.70–2.05)*	2.81 (2.50–3.17)*
OR Adj	Reference	1.22 (1.03–1.45)**	1.42 (1.19–1.69)**	1.55 (1.26–1.91)*
Composite morbidity *N* (%)	974 (1.09)	4,183 (1.93)	4,314 (4.12)	1,702 (7.06)
OR Unadj	Reference	1.78 (1.66–1.91)*	3.89 (3.63–4.18)*	6.88 (6.35–7.45)*
OR Adj	Reference	1.71 (1.51–1.94)*	2.16 (1.91–2.46)*	2.57 (2.23–2.95)*

^a^ Variables considered and adjusted in the multivariate analysis were: underlying medical conditions [congestive heart failure within 30 days prior to surgery, severe COPD, acute renal failure (pre-operative), bleeding disorders, cancer, ascites within 30 days prior to surgery], preoperative treatments (dialysis, ventilation, chemotherapy, radiation therapy, and ASA classification.

* *p* < 0.0001.

** *p* < 0.05.

Furthermore, patients were categorized according to their age and BMI into nine groups. Table 5 describes the incidence of surgery outcomes as numbers and percentages in the nine groups. Group 7 (older age and lower BMI) had the highest incidence of the described outcomes (mortality and morbidity), while the young age groups (1–3) had the lowest incidence of mortality and morbidity. Supplementary Figure S1 in the supplement shows the incidence of mortality and composite morbidity in the nine groups. Group 7 had the highest incidence of mortality (2.85%) and composite morbidity (8.90%). Both, group 8 (age > 70 and BMI 18.5–25) and group 9 (age > 70 and BMI > 25) showed a higher incidence of mortality and composite morbidity compared to groups 1–6, but lower than group 7.

**Table 5:** Incidence of outcome in the 9 different age BMI groups. Age and BMI groups were divided into 9 groups. Group 1 age < 50 and BMI < 18.5, group 2 age < 50 and BMI 18.5–25, group 3 age < 50 and BMI > 25, group 4 age 50–70 and BMI < 18.5, group 5 age 50–70 and BMI 18.5–25, group 6 age 50–70 and BMI > 25, group 7 age > 70 and BMI < 18.5, group 8 age > 70 and BMI 18.5–25, group 9 age > 70 and BMI > 25.

	Age and BMI Groups								
	1	2	3	4	5	6	7	8	9
Mortality *N* (%)	3 (0.17)	11 (0.03)	78 (0.04)	9 (0.79)	77 (0.32)	222 (0.18)	25 (2.85)	235 (1.55)	417 (0.91)
Wound outcome *N* (%)	10 (0.56)	152 (0.43)	839 (0.45)	15 (1.32)	269 (1.12)	1,356 (1.08)	22 (2.51)	280 (1.84)	880 (1.91)
Cardiac outcome *N* (%)	3 (0.17)	9 (0.03)	71 (0.04)	3 (0.26)	67 (0.28)	280 (0.22)	12 (1.37)	137 (0.90)	355 (0.77)
Respiratory outcome *N* (%)	18 (1.101)	84 (0.24)	518 (0.28)	26 (2.29)	204 (0.85)	975 (0.77)	39 (4.45)	365 (2.40)	1,123 (2.44)
Urinary outcome *N* (%)	4 (0.22)	11 (0.03)	117 (0.06)	4 (0.35)	48 (0.20)	304 (0.24)	2 (0.23)	72 (0.47)	317 (0.69)
CNS outcome *N* (%)	0 (0.00)	5 (0.01)	20 (0.01)	0 (0.00)	11 (0.05)	56 (0.04)	5 (0.57)	47 (0.31)	86 (0.19)
Thromboembolism *N* (%)	3 (0.17)	49 (0.14)	305 (0.17)	3 (0.26)	53 (0.22)	389 (0.31)	8 (0.91)	91 (0.60)	286 (0.62)
Sepsis *N* (%)	13 (0.73)	132 (0.37)	782 (0.42)	25 (2.20)	290 (1.21)	1,507 (1.20)	33 (3.77)	430 (2.83)	1,354 (2.94)
Return to OR *N* (%)	27 (1.51)	287 (0.81)	1,304 (0.71)	24 (2.11)	295 (1.23)	1,321 (1.05)	27 (3.08)	302 (1.99)	783 (1.70)
Composite morbidity *N* (%)	37 (2.08)	354 (1.00)	2,108 (1.14)	59 (5.20)	713 (2.98)	3,669 (2.91)	78 (8.90)	1,015 (6.67)	3,140 (6.82)

## Discussion

Laparoscopic cholecystectomy is generally considered a safe surgical procedure with a low risk of complications, a notion supported by our study's results, which revealed a very low incidence of mortality and morbidity in patients undergoing this procedure.

We evaluated the influence of age, BMI, and their combined effect on mortality and morbidity outcomes among laparoscopic cholecystectomy patients. Our findings showed a positive association between older age and both mortality and morbidity, with the adjusted risk for mortality increasing 16-fold in the older age group (those over 80 years old) and the adjusted risk for composite morbidity increasing 2.3-fold with advancing age. This observation is consistent with the well-established understanding that older age is a significant risk factor for postoperative morbidity and mortality, as reported in multiple studies (8, 9, 10, 11, 12, 13, 14). Our findings are also in line with a recent retrospective study by Enami et al. (8), which found that older age (≥79 years) was an independent predictor of postoperative complications after laparoscopic cholecystectomy. Similarly, Harino et al. (9) found that older age was associated with a higher risk of needing to convert a laparoscopic cholecystectomy to an open surgery.

On the other hand, we observed a significant protective effect of higher BMI against both mortality and composite morbidity. Furthermore, our analysis indicated that a higher age/BMI score elevated the adjusted risk for mortality and composite morbidity, although age remained the more prominent risk factor. Interestingly, we found that higher age/BMI score was a significant predictor of return to the operating room and the development of thromboembolism, while age alone was not. Our findings may challenge the conventional belief that obesity increases the risk of surgery, potentially leading to worse outcomes. This is supported by previous research, such as a study by Mullen et al. (4) on patients undergoing intra-abdominal cancer surgery. Mullen et al. found that obesity was only associated with superficial wound infections but not mortality, and that mild obesity may even be protective against mortality (4). Likewise, in a recent retrospective study on mortality and morbidity after laparoscopic cholecystectomy, Enami et al. (8) reported that obesity was not a risk factor for postoperative complications. This protective effect of BMI is consistent with the "obesity paradox," which is the observation that overweight and mildly obese people seem to have better outcomes than normal-weight people in certain situations, such as critical illness, congestive heart failure, and advanced coronary artery disease (15, 16, 17, 18). One possible explanation for the obesity paradox is that obese people have a low-grade chronic inflammation that primes their immune system to respond more effectively to the stress of surgery. This is in contrast to underweight people, who may have difficulty responding to stress due to underlying metabolic dysfunction and immunosuppression. Overall, our findings suggest that we need to reconsider the conventional wisdom that obesity increases the risk of surgery. More research is needed to understand the complex relationship between obesity and surgical outcomes.

We postulate that the association between lower BMI and the risk of mortality and morbidity observed in our study could be due to malnutrition. Rudasill et al. (19) investigated the role of malnutrition in morbidity and mortality in laparoscopic cholecystectomy and found that it was associated with an increased risk of morbidity and mortality. Similarly, Castillo-Angeles et al. (20) investigated the association of frailty with morbidity and mortality in 882,929 patients undergoing emergency general surgery and found that frailty was significantly associated with mortality. This finding may explain the higher risk of mortality and morbidity in our older patients with lower BMI.

Moreover, sarcopenia may explain the protective effect of higher BMI in older adults. A meta-analysis found that sarcopenia was associated with a higher risk of major adverse cardiovascular events in elderly patients with coronary artery disease who underwent cardiac surgery (21). Similarly, sarcopenia was associated with an increased risk of postoperative complications in patients undergoing gastrointestinal surgery (22). However, caution is needed, as the relationship between sarcopenia and BMI is complex (23).

The conclusions of this study must be considered along with its limitations, including those inherent to retrospective designs and the use of registry data. Specifically, the NSQIP data does not collect information on the underlying disease or condition that led to the surgery. Therefore, it is possible that our results may be confounded by the different indications for surgery in the study population. Additionally, our analysis focused only on laparoscopic cholecystectomy, a surgical procedure with a generally low risk of mortality and morbidity. Our findings may potentially apply to other low-risk surgical procedures, such as hernia repair, but may differ for surgeries with higher risk.

## Conclusion

Older age, especially accompanied by a low BMI, appears to increase the risk of mortality and morbidity post-surgery in laparoscopic cholecystectomy patients, while paradoxically, a higher BMI seems to be protective. Our hypothesis is that a lower BMI, perhaps secondary to malnutrition, can carry a greater risk of surgery complications for the elderly. Age/BMI is strongly and positively associated with mortality and morbidity and could be used as a new scoring system for predicting outcomes in patients undergoing surgery. Nevertheless, laparoscopic cholecystectomy remains a very safe procedure with relatively low complication rates.

## Data Availability

The data for this study was obtained from the American College of Surgeons National Surgical Quality Improvement Program (ACS NSQIP) Participant Use Data File. Additional results can be found in the Supplementary material.
