# Supplementary material for: Combined effect of age and body mass index on postoperative mortality and morbidity in laparoscopic cholecystectomy patients
Source: Front Surg. 2023 Nov 23;10:1243915. doi: 10.3389/fsurg.2023.1243915 (PMC10701421; doi:10.3389/fsurg.2023.1243915)
Supplement: Supplementary file 1 [file Table1.docx]

| **Table S1:** Baseline characteristics of the four age groups. Results are presented as mean (SD) for continuous variables and N (number) and % for categorical variables. ^[[1]](#footnote-1)^  **Age Groups** | | | | | |
| --- | --- | --- | --- | --- | --- |
| **Variables** | **< 40 years** | **40-<60 years** | **60-<80 years** | **>80 years** | **P value** |
| **Number** | 142901 | 161192 | 111537 | 19422 |  |
| **Age Mean (SD)** | 30.05 (5.81) | 49.61 (5.72) | 67.97 (5.50) | 84.14 (3.25) | <0.0001 |
| **Height (inch) Mean (SD)** | 64.636 (3.74) | 65.28 (3.96) | 65.31 (4.09) | 64.55 (4.19) | <0.0001 |
| **Weight (lb) Mean (SD)** | 194.74 (55.78) | 195.49 (51.30) | 184.97 (44.33) | 163 (36.81) | <0.0001 |
| **BMI Mean (SD)** | 32.68 (8.57) | 32.19 (7.76) | 30.45 (6.67) | 27.50 (5.47) | <0.0001 |
| **OP time (min) Mean (SD)** | 64.88 (40.96) | 70.61 (50.77) | 75.28 (57.80) | 74.29 (49.79) | <0.0001 |
| **Sex (female) N (%)** | 117112 (81.99) | 111589 (69.26) | 65434 (58.70) | 10886 (56.10) | <0.0001 |
| **Race (white) N (%)** | 94445 (66.09) | 109160 (67.72) | 79756 (71.51) | 14782 (76.11) | <0.0001 |
| **BMI >30 N (%)** | 35591 (24.91) | 46288 (28.72) | 37860 (33.94) | 7373 (37.96) | <0.0001 |
| **Smoking N (%)** | 29863 (20.90) | 30957 (19.21) | 12452 (11.16) | 668 (3.44) | <0.0001 |
| **Diabetes N (%)** | 5212 (3.65) | 19926 (12.36) | 26007 (23.32) | 4131 (21.27) | <0.0001 |
| **Hypertension N (%)** | 10396 (7.27) | 52457 (32.54) | 69779 (62.56) | 14908 (76.76) | <0.0001 |
| **Systemic sepsis in the previous 48 hours N (%)** | 8599 (6.02) | 10710 (6.64) | 10759 (9.65) | 3003 (15.46) | <0.0001 |
| **Steroid use N (%)** | 1409 (0.99) | 3324 (2.06) | 3596 (3.22) | 678 (3.49) | <0.0001 |
| **ASA IV/V N (%)** | 660 (0.46) | 2358 (1.46) | 4957 (4.45) | 2098 (10.82) | <0.0001 |
| **Inpatient N (%)** | 50250 (35.16) | 58132 (36.06) | 50029 (44.85) | 12642 (65.09) | <0.0001 |
| **Transfusion N (%)** | 216 (0.15) | 234 (0.15) | 361 (0.32) | 135 (0.70) | <0.0001 |
| **Wound infection N (%)** | 1944 (1.36) | 4511 (2.80) | 6546 (5.87) | 1822 (9.38) | <0.0001 |
| **Dyspnea N (%)** | 2426 (1.70) | 6017 (3.73) | 7946 (7.12) | 1916 (9.87) | <0.0001 |
| **Congestive heart failure in the previous 30 days N (%)** | 87 (0.06) | 453 (0.28) | 1133 (1.02) | 528 (2.72) | <0.0001 |
| **COPD N (%)** | 256 (0.18) | 3082 (1.91) | 6232 (5.59) | 1481 (7.63) | <0.0001 |
| **Ascites N (%)** | 81 (0.06) | 241 (0.15) | 253 (0.23) | 53 (0.27) | <0.0001 |
| **Renal failure N (%)** | 54 (0.04) | 191 (0.12) | 374 (0.34) | 87 (0.45) | <0.0001 |
| **Bleeding disease N (%)** | 1036 (0.72) | 2643 (1.64) | 5308 (4.76) | 1881 (9.68) | <0.0001 |
| **Disseminated cancer N (%)** | 150 (0.10) | 982 (0.62) | 1477 (1.32) | 259 (1.33) | <0.0001 |

**Table S2:** Baseline characteristics of the BMI categories. Results are presented as mean (SD) for continuous variables and N (number) and % for categorical variables. ^[[2]](#footnote-2)^

| BMI Groups | | | | |
| --- | --- | --- | --- | --- |
|  | **1** | **2** | **3** | **P value** |
| Number | 78421 | 127112 | 229519 |  |
| Age mean (SD) | 51.28 (19.18) | 51.89 (17.44) | 47.44 (16.12) | <0.0001 |
| Sex (female) N (%) | 56960 (72.69) | 80164 (63.10) | 167897 (73.19) | <0.0001 |
| Height (inch) Mean (SD) | 64.99 (3.82) | 65.43 (3.99) | 64.84 (3.95) | <0.0001 |
| Weight (lb) Mean (SD) | 135.28 (20.43) | 168.55 (22.48) | 222.69 (46.84) | <0.0001 |
| BMI Mean (SD) | 22.43 (2.03) | 27.57 (1.42) | 37.14 (6.64) | <0.0001 |
| OP time (min) Mean (SD) | 66.71 (52.35) | 68.03 (49.94) | 72.38 (48.90) | <0.0001 |
| Race (white) N (%) | 53036 (67.63) | 87972 (69.21) | 157135 (68.46) | <0.0001 |
| Smoking N (%) | 15094 (19.25) | 21104 (16.60) | 37742 (16.44) | <0.0001 |
| Diabetes N (%) | 6013 (7.67) | 13780 (10.84) | 35483 (15.46) | <0.0001 |
| Hypertension N (%) | 20961 (26.73) | 41796 (32.88) | 84783 (36.94) | <0.0001 |
| Systemic sepsis in the previous 48 hours N (%) | 5174 (6.60) | 9718 (7.65) | 18179 (7.92) | <0.0001 |
| Steroid use N (%) | 2108 (2.69) | 2609 (2.05) | 4290(1.87) | <0.0001 |
| ASA IV/V N (%) | 2152 (2.75) | 2634 (2.08) | 5287 (2.31) | <0.0001 |
| Inpatient N (%) | 20262 (38.59) | 49638 (39.05) | 91153 (39.71) | <0.0001 |
| Transfusion N (%) | 322 (0.41) | 257 (0.20) | 367 (0.16) | <0.0001 |
| Wound infection N (%) | 2372 (3.02) | 4764 (3.75) | 7687 (3.35) | <0.0001 |
| Dyspnea N (%) | 2815 (3.59) | 4283 (3.37) | 11207 (4.88) | <0.0001 |
| Congestive heart failure in the previous 30 days N (%) | 412 (0.53) | 576 (0.45) | 1213 (0.53) | 0.0069 |
| COPD N (%) | 2585 (3.30) | 3097 (2.44) | 5369 (2.34) | <0.0001 |
| Ascites N (%) | 196 (0.25) | 199 (0.16) | 233 (0.10) | <0.0001 |
| Renal failure N (%) | 142 (0.18) | 203 (0.16) | 361 (0.16) | NS |
| Bleeding disease N (%) | 2184 (2.78) | 3415 (2.69) | 5269 (2.30) | <0.0001 |
| Disseminated cancer N (%) | 923 (1.18) | 967 (0.76) | 978 (0.43) | <0.0001 |

**Figure S1:** Incidence of mortality and composite morbidity according Age BMI groups. The cohort was divided into 9 groups according to age and BMI. Group 1 age<50 and BMI <18.5, group 2 age<50 and BMI 18.5-25, group 3 age<50 and BMI >25, group 4 age 50-70 and BMI <18.5, group 5 age 50-70 and BMI 18.5-25, group 6 age 50-70 and BMI >25, group 7 age>70 and BMI <18.5, group 8 age>70 and BMI 18.5-25, group 9 age>70 and BMI >25.

1. OP time: operation time; ASA: American Society of Anesthesiologists; COPD: chronic obstructive pulmonary disease. [↑](#footnote-ref-1)
2. OP time: operation time; ASA: American Society of Anesthesiologists; COPD: chronic obstructive pulmonary disease. [↑](#footnote-ref-2)
